# Late Deep Infections Complicating Percutaneous Pinning of Supracondylar Humerus Fractures

**DOI:** 10.1155/2021/7915516

**Published:** 2021-09-30

**Authors:** Achraf H. Jardaly, Ketrick LaCoste, Shawn R. Gilbert, Michael J. Conklin

**Affiliations:** ^1^University of Alabama at Birmingham, Department of Orthopaedics, Birmingham, AL, USA; ^2^Children's Hospital of Alabama, Department of Pediatric Orthopaedics, Birmingham, AL, USA

## Abstract

**Objectives:**

Complications following treatment of supracondylar humerus fractures are typically seen shortly postoperatively. Late complications occurring years after percutaneous pinning are rare but can be indolent and have permanent sequelae. We present cases of children presenting with late deep infections to discuss their diagnosis and treatment.

**Methods:**

After institutional review board approval, we retrospectively reviewed records of three children who developed deep infections at least one year after percutaneous pinning of their supracondylar humerus fracture. Patient details and outcomes were analyzed. Radiographs and magnetic resonance imaging were reviewed along with each patient's clinical course and treatment.

**Results:**

We report 3 cases of osteomyelitis and/or septic arthritis presenting at least one year after supracondylar humerus fractures treated with closed reduction and percutaneous pinning. The patients required several irrigation and debridement procedures with placement of antibiotic beads in addition to a prolonged course of antibiotics.

**Conclusion:**

Delayed deep infections can occur after closed reduction and percutaneous pinning of supracondylar humerus fractures in children. Vigilance is required to diagnose and treat such occurrences, and prolonged follow-up is needed to monitor for recurrent or intractable infections.

## 1. Introduction

Supracondylar fractures of the distal humerus are the most common pediatric elbow fractures. For nondisplaced fractures (type I), immobilization and casting are used. Several treatment modalities are available for displaced fractures. Closed reduction and immobilization with the collar-and-cuff technique as described by Blount can yield satisfactory results, but surgical fixation, namely, with open or closed reduction and percutaneous pinning (CRPP), is more commonly used [1, 2].

CRPP is a safe, effective procedure with a low incidence of complications. In their review of 622 children surgically managed for supracondylar humerus fractures, Bashyal et al. showed that the most common complication is pin migration, occurring in 1.8% of patients [3]. Infections are rare, with superficial infections occurring in 1% of children and deep infections in 0.2%. Most complications occur early, diagnosed within weeks to months of CRPP. In our review of the literature, we found only a single case of late-onset osteomyelitis in the setting of CRPP for supracondylar fractures [4]. Despite being rare, awareness of delayed deep infections is necessary, as they can have permanent sequelae if not adequately diagnosed and managed. Here, we report a case series of three such infections occurring at least one year after supracondylar fracture pinning and discuss their management. Patients and their guardians agreed to publishing details of their case.

## 2. Case Presentation

### 2.1. Case 1

**Figure 1:**
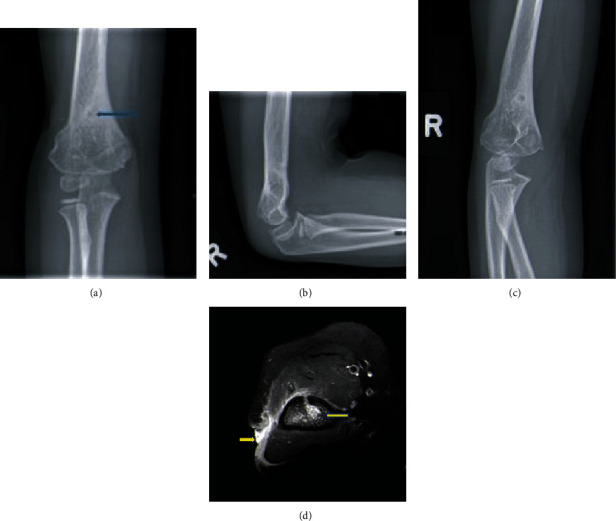
(a–c) AP, lateral, and oblique MRI of the right elbow with a subtle area of lucency (arrows) in the metaphysis. (d) Axial T1 postcontrast MRI with an enhancing lesion (large arrow) in the bone with anterior cortical disruption and enhancement anterior to the humerus extending to the lateral sinus tract (small arrow).

A five-year-old female was treated at a different institution with CRPP for a right supracondylar humerus fracture. One week postoperation, she developed acute pin-site infection that seemingly resolved with a short course of oral cephalexin. Approximately one year later, she presented with purulent drainage from the previously healed wound and was found to have a sinus tract to the bone of her distal humerus. The patient was treated with IV antibiotics and received 10 day courses of amoxicillin/clavulanic acid and trimethoprim/sulfamethoxazole in order to treat several other episodes of purulent drainage from her wound over the next 5.5 months. The patient experienced no significant clinical improvement and presented to our institution 16 months after pin removal. Cultures of the drainage grew methicillin-sensitive *Staphylococcus aureus* (MSSA). Laboratory investigations including inflammatory markers and WBC were unremarkable. Radiographs revealed a lucency with surrounding sclerosis in the distal humeral metaphysis (Figures 1(a)–1(c)), and MRI disclosed an enhancing lesion with a lateral sinus tract (Figure 1(d)). She then underwent pin-tract I&D, bone curettage, and sinus excision. Operative cultures also grew MSSA. She was given vancomycin via a PICC line for 4 weeks postoperation. She was switched to oral cephalexin and clindamycin for 6 weeks and maintained on cephalexin for 6 months. On exam at that time, her wound had healed, her elbow ROM was normal, and her laboratory studies were unremarkable. Antibiotics were discontinued.

Thirteen months later, the patient returned with a 1-month history of arm pain. Radiographs showed a 1.5 cm lucency in the distal humeral metaphysis with periosteal reaction. The patient was then placed on cephalexin and clindamycin before undergoing repeat I&D with placement of antibiotic-impregnated calcium phosphate bone cement. She was maintained on cephalexin for 12 months. Approximately 2 months after stopping cephalexin, her infection recurred with pain, swelling, warmth, and erythema of the right elbow with associated low-grade fevers following trauma from a fall. The patient was then treated more extensively with I&D with wide curettage and placement of antibiotic methyl methacrylate beads (Simplex P, Howmedica Osteonics Corp., Mahwah, NJ, USA). This procedure was repeated 4 days later with bead exchange and once more after 3 weeks with placement of resorbable vancomycin containing calcium sulfate cement. She received IV cefazolin for 5 days and oral cephalexin for 12 months. She healed and has remained asymptomatic for over 1 year after stopping antibiotics.

### 2.2. Case 2

**Figure 2:**
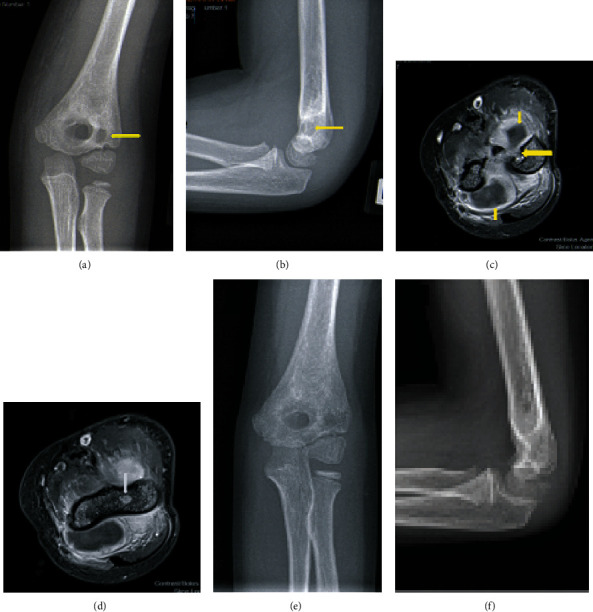
(a, b) AP and lateral x-rays showing a distal humeral lucency concerning for chronic osteomyelitis. (c, d) Axial T1 post contrast MRI. (c) The large arrow denotes nidus of infection. (d) The large arrow denotes anterior cortical disruption. The small arrows denote purulent fluid collection in elbow joint. (e) and (f) are radiographs one year after irrigation and debridement.

A four-year-old male presented with a left type III supracondylar fracture which was treated with CRPP and casting. The pins were removed 3 weeks after surgery with no complications. Twenty-eight months later, the patient presented with atraumatic left elbow pain. He was tender over the medial aspect of the elbow and exhibited some pain with ROM. X-rays showed a lucency over the distal humerus (Figures 2(a) and 2(b)), suspected to be a nidus for chronic osteomyelitis. MRI demonstrated an effusion and humeral changes (Figures 2(c) and 2(d)) consistent with osteomyelitis and septic arthritis. The patient underwent aspiration of his elbow and I&D with placement of antibiotic beads. Intraoperative cultures grew methicillin-resistant *Staphylococcus aureus* and *Streptococcus pyogenes*. Repeat I&D with antibiotic bead exchange was performed 3 weeks later. The patient was placed on intravenous ampicillin for 24 hours and was discharged on amoxicillin and trimethoprim/sulfamethoxazole for 6 weeks and was maintained on trimethoprim/sulfamethoxazole for 6 months. At his 6-month follow-up, his exam was unremarkable. His infection completely resolved, and he did not have recurrence after finishing his antibiotic regimen. His last follow-up was 6 months after cessation of antibiotics (13 months after his last I&D), and he is still being observed.

### 2.3. Case 3

A four-year-old male underwent CRPP for a left supracondylar humerus fracture at a different institution. He developed an acute pin-site infection treated with oral doxycycline before pin removal. Three years later, the patient presented to our institution for atraumatic left elbow pain and swelling with fever. The patient's parents endorsed multiple similar episodes occurring intermittently over the last 3 years, with complete resolution of symptoms between episodes. MRI showed a nidus of osteomyelitis in the distal humerus with a tract communicating with the elbow and an effusion characteristic of septic arthritis. He underwent elbow aspiration and I&D with placement of methyl methacrylate/vancomycin beads (Simplex P, Howmedica Osteonics Corp., Mahwah, NJ, USA) followed by a repeat I&D with bead exchange 4 days later. Intraoperative cultures grew MSSA. Therefore, cefazolin was administered via PICC line for 2 weeks. The patient then had a repeat I&D with antibiotic bead removal and insertion of resorbable antibiotic beads 2 weeks later and was started on cephalexin for 6 months. Upon completion of the treatment regimen, the patient had healed. Physical exam was unremarkable. He was followed for 18 months and did not develop any signs of recurrent infection.

## 3. Discussion

CRPP is a common procedure for the treatment of displaced pediatric supracondylar fractures with high efficacy and extremely low rate of complications [1, 3, 5]. Infection is a rare complication of CRPP, but most are superficial tissue infections. In even rarer cases, a minor pin track infection can develop from local extension of superficial infections. However, infections of this nature usually take place in the early postoperative period [3].

Delayed pin track infections are a rare occurrence in any orthopedic procedure, even more so in CRPP. They have been reported in fractures treated with external fixation or skeletal traction, but there is little mention in the literature of delayed pin track infections in supracondylar fractures treated with CRPP [6–10]. Pulos and Carrigan reported a case of late onset osteomyelitis in an 11-year-old male presenting with vague symptoms 4 years following an uneventful CRPP for the treatment of a supracondylar fracture, with maximum symptomatic presentation in the form of elbow fullness and tingling at 6 years following CRPP, followed shortly by the development of a draining wound [4].

While a draining wound in this scenario necessitates culture of the drainage and imaging for the investigation of potential osteomyelitis, the presentation of a delayed pin-site infection can be varied and much more indolent [11]. Case 2 had only vague atraumatic elbow pain without any sort of draining wound or skin changes on initial examinations. The range of symptomatic presentations in this case series suggests that the diagnosis of delayed pin track infection with late-onset osteomyelitis should be suspected in the setting of pain, swelling, or drainage at the site of a previous fracture. Imaging studies aid the diagnosis of infection. Pin tracks typically result in a sclerotic appearance due to deposition of new bone, and an area of lucent ring sequestrum is indicative of bone destruction and is highly suggestive of infection [6, 12]. Preoperative advanced imaging like CT and MRI further aid the diagnosis of infection and are useful for isolating possible foci of infection in surgical planning.

Though the presentation of delayed pin track infections can appear nonspecific and suggest a less urgent pathology, they can progress to severe or recurrent infections. Case 1 had a recalcitrant infection with several recurrences over a 2-year time period before the resolution of the pin track infection. Also, cases 2 and 3 had concomitant septic arthritis. The potential for serious complications associated with classic hematogenous osteomyelitis also exists in delayed pin track infections. Therefore, patients should be managed expeditiously to prevent significant morbidity from the extension of these infections. Treatment of deep infections should involve obtaining cultures before administration of antibiotics with adjustment of antibiotic coverage after culture and sensitivity testing, surgical I&D, and fluid aspiration in the case of a septic joint [11, 13–15]. The possibility for persistent infections, as in these cases, made us more aggressive in treating subsequent similar infections with planned early repeat debridement and prolonged antibiotics for 6 months. Careful postoperative monitoring should include follow-up even after patients complete their antibiotic regimens.

In conclusion, late deep infections are a rare complication of CRPP, but surgeons must consider them as a possibility in patients who have undergone this procedure to allow for prompt diagnosis and adequate treatment should they occur.

## Abbreviations

AP: Anteroposterior
CRPP: Closed reduction and percutaneous pinning
CT: Computerized tomography
I&D: Irrigation and debridement
IV: Intravenous
MRI: Magnetic resonance imaging
MSSA: Methicillin-sensitive *Staphylococcus aureus*
PICC: Peripherally inserted central catheter
ROM: Range of motion
WBC: White blood cells.
